# Exploration of the rhizosphere microbiome of native plant *Ceanothus velutinus* – an excellent resource of plant growth-promoting bacteria

**DOI:** 10.3389/fpls.2022.979069

**Published:** 2022-12-15

**Authors:** Jyothsna Ganesh, Vishal Singh, Katherine Hewitt, Amita Kaundal

**Affiliations:** Department of Plants, Soils, and Climate, College of Agriculture and Applied Sciences, Utah State University, Logan, UT, United States

**Keywords:** rhizosphere, plant growth promoting rhiobacteria (PGPR), native plants, *Ceanothus velutinus*, Intermountain West (US), snowbrush ceanothus

## Abstract

Continuous demand for an increase in food production due to climate change and a steady rise in world population requires stress-resilient, sustainable agriculture. Overuse of chemical fertilizers and monoculture farming to achieve this goal deteriorated soil health and negatively affected its microbiome. The rhizosphere microbiome of a plant plays a significant role in its growth and development and promotes the plant’s overall health through nutrient uptake/availability, stress tolerance, and biocontrol activity. The Intermountain West (IW) region of the US is rich in native plants recommended for low water use landscaping because of their drought tolerance. The rhizosphere microbiome of these native plants is an excellent resource for plant growth-promoting rhizobacteria (PGPR) to use these microbes as biofertilizers and biostimulants to enhance food production, mitigate environmental stresses and an alternative for chemical fertilizer, and improve soil health. Here, we isolated, purified, identified, and characterized 64 bacterial isolates from a native plant, *Ceanothus velutinus*, commonly known as snowbrush ceanothus, from the natural habitat and the greenhouse-grown native soil-treated snowbrush ceanothus plants. We also conducted a microbial diversity analysis of the rhizosphere of greenhouse-grown native soil-treated and untreated plants (control). Twenty-seven of the 64 isolates were from the rhizosphere of the native region, and 36 were from the greenhouse-grown native soil-treated plants. These isolates were also tested for plant growth-promoting (PGP) traits such as their ability to produce catalase, siderophore, and indole acetic acid, fix atmospheric nitrogen and solubilize phosphate. Thirteen bacterial isolates tested positive for all five plant growth-promoting abilities and belonged to the genera *Pantoea*, *Pseudomonas*, *Bacillus*, and *Ancylobacter*. Besides, there are isolates belonging to the genus *Streptomyces*, *Bacillus*, *Peribacillus*, *Variovorax*, *Xenophilus*, *Brevundimonas*, and *Priestia*, which exhibit at least one of the plant growth-promoting activities. This initial screen provided a list of potential PGPR to test for plant health improvement on model and crop plants. Most of the bacterial isolates in this study have a great potential to become biofertilizers and bio-stimulants.

## 1 Introduction

Climate change has caused a drastic loss to crop production in the first decade of the 21^st^ century, with a 3.8 and 5.5% decline in globally for maize and wheat production, respectively (Lobell et al., 2011). Studies showed that each degree Celsius increase in temperature could lead to a 5% reduction in yield (Lobell et al., 2011). On top of that, to feed a continuously growing world population of ~9 billion by 2050, production must increase by 50% (Alexandratos and Bruinsma, 2012). Environmental stresses such as salinity, drought, and rising carbon dioxide levels have posed a significant threat to sustainable agriculture (Tuomisto et al., 2017). While soil salinity diminishes growth and development by affecting morphological, physiological, and biochemical aspects of plant growth (Gupta and Huang, 2014), drought is another significant environmental stress that harms plants and declines the productivity of crops (Bodner et al., 2015). It has been anticipated that by 2050 drought will be the lead cause of growth problems in plants due to the increase in climate change (Vinocur and Altman, 2005). A combination of stresses during the crop growth period has led to a severe loss of productivity (Mahalingam, 2015). Anthropogenic abuses and extreme weather conditions due to climate change negatively affect soil health and the microbiome.

The soil microbiome is essential for soil health and affects plant growth and development (Timmis and Ramos, 2021). The rhizosphere is the soil zone under the direct influence of the roots of plants. Microorganisms in the rhizosphere communicate with plants’ roots and influence their functions and play a significant role in plant health, nutrition, and yield (Habibi et al., 2014). The rhizosphere soil shelters various beneficial microbes, such as plant growth-promoting rhizobacteria (PGPR), arbuscular mycorrhizal fungi (AMF), microparasitic fungi, and protozoa. PGPRs are beneficial microorganisms that colonize around the roots and reside in the rhizosphere region of plants (Kumari et al., 2018). They enhance the tolerance of biotic and abiotic stresses such as salinity, heavy metals, drought, etc. (Selvakumar et al., 2012). The PGPRs directly promote a plant’s growth either by secreting plant hormones such as auxins (Indole acetic acid- IAA), cytokinins, gibberellins (GA_3_), and ethylene (Chabot et al., 1996; Bent et al., 2001) or enhancing nutrient availability by nitrogen fixation, solubilization of phosphate or other minerals such as potassium and zinc, production of ACC- deaminase enzyme, and siderophore production (Ahmad et al., 2008; Habibi et al., 2014). They indirectly promote plants’ growth by suppressing infection by pathogenic bacteria, fungi, nematodes, and viruses or acting as biocontrol agents (Barea et al., 2005). A plant’s root architecture greatly stimulates its microbiome and provides a unique ecological niche for microbes to recruit to the rhizosphere and inside roots (Hartmann et al., 2009).

Geochemically distinct bulk soils influence the bacterial diversity in the rhizosphere and endophytic interactions in Arabidopsis (Lundberg et al., 2013). A recent finding has revealed that the native microbiota of the resistant tomato variety suppresses *Ralstonia solanacearum*-disease development in the susceptible variety (Kwak et al., 2018). Native plants are a great source of such potentially beneficial native microbiota unknown to the current agricultural system. According to the Wildlife federation, a plant is considered native if it has occurred naturally in a particular region, ecosystem, or habitat without human introduction. Native plants have formed symbiotic relationships with native wildlife over thousands of years and offer the most sustainable habitat. Various studies have reported and suggested using drought-tolerant native plants for low-water landscapes (McKinney, 2002; Yilmaza and Yilmazb, 2009; Sun et al., 2019). The Intermountain West region (IW) of the US is rich in drought-tolerant native plants, both woody and herbaceous, and few of them have been recommended to be used in water-efficient landscaping (Kratsch, 2011; Rosentreter et al., 2017). The microbiome of these plants has not been explored yet. Investigation of rhizospheric microbiomes of these plants can open new avenues to enhance the knowledge about their potential to mitigate climate change by supporting resilient agriculture, replacing chemical fertilizers with biofertilizers, and sustaining soil microbiomes. *Ceanothus velutinus*, commonly known as snowbrush ceanothus is an evergreen plant native to western North America. Its habitat ranges from British Colombia and Alberta to Utah, Colorado, and California. It is an ornamental and medicinal plant, as Native Americans used it to treat pain, flu, and gonorrhoea (Francis, 2004). It is an actinorhizal plant which form nodules by actinobacteria *Frankia* and capable of nitrogen fixation and is reported to be heat and drought-tolerant (Zavitkovski, 1966; Jeong and Myrold, 2001; Stein et al., 2010).

In this study, we isolated potential plant growth-promoting bacteria from the rhizosphere of snowbrush ceanothus and characterized them for a few plant growth-promoting traits. Rhizospheric microbial diversity from native soil-treated plants in the greenhouse was assessed using a metagenomic analysis approach. The effect of native soil on the growth and development of greenhouse-grown snowbrush ceanothus plants was evaluated.

## 2 Materials and methods

### 2.1 Collection of rhizospheric soil samples from the native habitat of snowbrush ceanothus

Rhizosphere soil samples from *Ceanothus velutinus* (snowbrush ceanothus) were collected from two elevations in the Tony Grove region near Logan, Utah- elevation 1950m AMSL (Above Mean Sea Level) (41°52’34” N- 111°34’20” W), and elevation 2289m AMSL (41°53’15” N - 111°36’4” W). Sample collected from the five plants from the elevation 1950m AMSL and three plants from the elevation 2289m AMSL. Snowbrush ceanothus is a big shrub with profound roots in the national forest. The methodology described elsewhere was used with slight modifications, where whole plant was not uprooted as described in protocol (McPherson et al., 2018). The primary root was exposed by digging 30” deep, and the lateral roots were excised with pruning scissors sterilized in 70% EtOH. The excised roots were collected in pre-sterilized 50ml tubes containing phosphate buffer with surfactant (Silwet-L77) and immediately stored on ice for transportation (6.33 g/L NaH_2_PO_4_, 8.5 g/L Na_2_HPO_4_ anhydrous, pH = 6.5, 200 μl/L Silwet-L77). The tubes with samples were shaken on a rotary shaker to separate the rhizosphere soil. The roots were removed, and the rhizosphere soil was collected by centrifuging tubes at 3000g for five minutes. The rhizosphere soil pellets were washed with phosphate buffer without surfactant and stored at 4°C.

### 2.2 Sample collection of rhizosphere soils from the greenhouse-treated plants

Two-months-old snowbrush ceanothus plants grown from cuttings were placed in 3.8 L pots with a soil mixture of 75% peat moss (Canadian Sphagnum peat moss, SunGro Horticulture Canada, Agawam, MA), 25% vermiculite (Therm-O-Rock West, Chandler, AZ), 0.89 kg.m^-3^ gypsum (92% calcium sulfate dihydrate, 21% calcium, 17% sulfur, athletic white sports field marking gypsum, Western Mining, and Minerals, Bakersfield, CA), 1.57 kg m^-3^ dolomitic lime (Lhoist North America, Salinas, CA), and 0.65 kg m^-3^ wetting agent (AquaGro G; Aquatrols^®^, Paulsboro, NJ), and controlled-release fertilizer (Chen et al., 2020). To see the effect of native soil on growth and development, we added 200 mL of native soil (ratio between soil substrate mixture and native soil is 19:1) collected from elevation 1950m AMSL (Treament1), and from elevation 2289m AMSL (Treatment 2), to three and two plants, respectively. Two plants were not inoculated with native soil and were considered as control. Snowbrush ceanothus is a difficult plant to propagate in nursery conditions by cuttings (Paudel et al., 2022). Cuttings are the preferred way to propagate plants to get uniform siblings compared to plants by seeds, so we had a limited number of plants for this study. This study was conducted for six months, and different observations were recorded at the end of the experiment. The number of secondary shoots and the amount of available Nitrate-nitrogen (NO_3_-N) content (Chen et al., 2020) were measured after six months of inoculation. 500 mL of tap water was poured slowly from the top with a tray below the pot to collect the leachate. After incubating it for 30 minutes, a few drops of the leachate were used to measure the nitrate- nitrogen (NO_3_-N) content (ppm) by a NO_3_-N meter (LAQUA Twin; Horiba, Kyoto, Japan). Five replicates were taken from each pot’s leachate to measure the NO_3_-N content. The rhizosphere soil was collected from the treated plants for PGPR isolation after six months of native soil treatments and stored at 4°C. Rhizosphere samples were also saved for metagenomics analysis and stored at -80°C. Sample collection was similar to the one followed for the native samples (McPherson et al., 2018). An analysis of variance (ANOVA) and Tukey-Kramer method for multiplicity at α = 0.05. was used to depict the statistical differences among control and treatments on SAS Studio (SAS Institute, Cary, NC).

### 2.3 Metagenomic analysis of the rhizosphere of snowbrush ceanothus plants

Microbial DNA was isolated from the rhizosphere soil of snowbrush ceanothus plants (control and treatment) from the greenhouse using Qiagen DNeasy PowerSoil Pro Kit. The isolated DNA was quantified by nanodrop 2000 (Thermo Scientific). The V4 variable region of 16S rRNA was sequenced. The 16S rRNA gene was amplified using the V4 variable region-specific primers 515F (5’-GTGCCAGCMGCCGCGGTAA-3’) and 806R (5’-GGACTACHVHHHTWTCTAAT-3’) (https://www.novogene.com/us-en/services/research-services/metagenome-sequencing/16s-18s-its-amplicon-metagenomic-sequencing/). The amplification reaction mixture (25 µl) contained 13 µl of water, 10 µl of Platinum Hot Start PCR Master Mix (MM) (Thermo Fisher), 0.5 µl of 10 mM forward and reverse primer each, and 1 µl of 5 ng/µl of the DNA. The polymerase chain reaction (PCR) conditions were as follows: an initial denaturation of three minutes at 94°C followed by 35 cycles of denaturation at 94°C for 45 seconds, annealing at 50°C for 60 seconds, an extension at 72°C for 60 seconds, and a final extension at 72°C for 10 minutes, and ending with an infinite hold at 4°C using the DNA engine dyad Peltier thermal cycler. The PCR products were diluted 50 times, and a secondary PCR reaction attached the indexes. The second reaction mixture consisted of 5 µl of MM, 2 µl of i5 index, 3 µl of i7 index, and 1 µl of the diluted PCR product. The PCR conditions are as follows: an initial denaturation step at 94°C for one minute followed by 15 cycles of denaturation at 94°C for 15 seconds, annealing at 64°C for 15 seconds, and an extension at 72°C for one minute, and a final extension at 72°C for three minutes using a DNA engine dyad Peltier thermal cycler (BIO-RAD). Once the indexes were attached, the samples were cleaned up with AMPureXP beads, using a 1:1 ratio. The PCR products were quantified by fluorometry, and quality was analyzed on the TapeStation. The samples were then pooled and sequenced on the MiSeq using a 2×250 paired-end sequencing chip size (Illumina).

The sequenced data were analyzed using various packages available in *R ver 4.0* (Team, 2021). Analysis of amplicon sequence variants (ASVs) was carried out by divisive amplicon denoising algorithm (DADA2) using R package *dada2* (Callahan et al., 2016), and the taxonomic classification was done. Relative abundance of different taxa was calculated as a ratio of read counts of the taxa to the total read counts. Alpha diversity plots were generated using the “plot_richness” function of an r package *phyloseq* (McMurdie and Holmes, 2013). Two different diversity indices “Shannon” and “Simpson” were used to estimate alpha diversity, a measure of diversity within a sample or community. While Shannon diversity index (Shannon, 1948) estimates the species richness alone, Simpson index considers species richness and relative abundance. (Simpson, 1949). Beta diversity plots showing between sample diversity were generated and plotted using PCA as an ordination method using the *phyloseq* package. Heat maps of the relative abundance of bacteria in different samples were generated using the “heatmap.2” function of gplots (version 3.1.1)

### 2.4 Isolation and purification of PGPR

The rhizosphere samples of five plants from elevation 1950m AMSL and three plants from the elevation 2289m AMSL were pooled together. The rhizosphere pellets were diluted to a 10:95 ratio of pellet to water, where 1 gram of soil was resuspended in 9.5 ml of sterilized water. It was then serially diluted in the ratio of 1:10 and 100 µl of the last three dilutions, viz. 10^-3^, 10^-4^, and 10^-5^ were spread plated onto the five media compositions viz., ¼ Nutrient Agar, ¼ Tryptic Soy Agar (SIGMA-ALDRICH), Yeast Mannitol Agar (SIGMA-Life Science), Minimal M9 Media (BD Difco), and Actinomycete Isolation Agar (SIGMA-ALDRICH) (**Tables S1, S2**). The plates were incubated at 28°C for 3-5 days. Once growth appeared, single colonies were selected based on their different visual characteristics such as color, texture, transparency, size, consistency, and any other distinct morphological trait, and selected colonies were purified by the streak plate method. The screening process was repeated three times, and the purified colonies were preserved as glycerol stocks at -80 °C.

### 2.5 PCR amplification, 16S rRNA sequencing, and BLAST

The isolated bacterial colonies were subjected to colony PCR to amplify the full length of the 16S rRNA region using the 27F (V1 region- 5′-AGAGTTTGATCCTGGCTCAG-3′) as the forward primer and 1492R (V9 region- 5′-TACGGYTACCTTGTTACGACTT-3′) as the reverse primer using DreamTaq DNA polymerase. The PCR program was as follows: 95°C initial denaturation for two minutes, 35 cycles of 95°C denaturation for 30 seconds, 54.3°C annealing for 30 seconds, and 72°C extension for one and a half minutes, followed by a final extension at 72°C for 10 minutes (Applied Biosystems- ProFlex PCR system). The PCR products were sequenced and results were run on a BLAST (Basic Local Alignment Search Tool) against a 16SrRNA database on NCBI to identify the bacterial species. The sequences were submitted to the GenBank on NCBI, and accession numbers are given in **Tables 1**, **2**.

**Table 1:** Bacterial characterization of the isolates from the rhizosphere of snowbrush ceanothus from the native region based on catalase production, siderophore, phosphate solubilization, nitrogen fixation, and IAA production.

S. No.	Code	Catalase	SP	PS	NF		IAA	BLAST	Accession numbers
					Media	*nif*H^+^			
1	GK_NR_127	+	–	–	–	ND	0	No Match	*-*
2	GK_NR_129	+	+++	–	–	ND	0.02 ± 0.06	*Streptomyces* sp.	OP407622
3	GK_NR_130	+	–	–	–	ND	0.17 ± 0.02	*Peribacillus* sp.	OP407623
4	GK_NR_131	–	–	–	–	ND	0.23 ± 0.09	*Neorhizobium* sp.	OP407624
5	GK_NR_133	+	+	+++	+++*	**-**	**33.52 ± 0.15**	*Pantoea* sp.	OP407625
6	GK_NR_136	+	++	–	–	ND	0	*Streptomyces* sp.	OP407626
7	GK_NR_139	++	–	–	–	ND	0	*Nocardia* sp.	OP407627
8	GK_NR_143	+	–	–	–	ND	2.41 ± 0.04	*Streptomyces* sp.	OP407628
9	GK_NR_144	+	+++	–	–	ND	0.66 ± 0.34	*Xenophilus* sp.	OP407629
10	GK_NR_145	+	–	+	–	ND	0.12 ± 0.12	*Streptomyces* sp.	OP407630
11	GK_NR_146	+	–	–	–	ND	0	*No match*	
12	GK_NR_149	+	+	–	–	ND	14.88 ± 0.11	*Brevibacterium* sp.	OP407631
13	GK_NR_150	+	+	–	–	ND	3.57 ± 0.04	*Leifsonia* sp.	OP407632
14	GK_NR_154	+	–	–	–	ND	0.03 ± 0.03	*Agromyces* sp.	OP407633
15	GK_NR_156	++	–	–	–	ND	0	*Staphylococcus* sp.	OP407634
16	GK_NR_162	+	–	+	–	ND	1.22 ± 0.06	*Streptomyces* sp.	OP407635
17	GK_NR_166	++	–	–	–	ND	0	*Streptomyces* sp.	OP407636
18	GK_NR_177	++	–	–	–	ND	0	*Promicromonospora* sp.	OP407637
19	GK_NR_179	+	–	–	–	ND	0	*Streptomyces* sp.	OP407638
20	GK_NR_180	+	–	–	–	ND	0	*Streptomyces* sp.	OP407639
21	GK_NR_182	+	–	++	–	ND	0	*Streptomyces* sp.	OP407640
22	GK_NR_186	+	–	+	–	ND	0.03 ± 0.04	*Streptomyces* sp.	OP407641
23	GK_NR_188	+	–	–	–	ND	0.35 ± 0.36	*Janthinobacterium* sp.	OP407642
24	GK_NR_194	+	+	–	+++	–	8.97 ± 0.45	*Pseudomonas* sp.	OP407643
25	GK_NR_195	++	–	–	–	ND	0	*No match*	*-*
26	GK_NR_196	+	–	–	–	ND	0	*Pedobacter* sp.	OP407644
27	GK_NR_197	+	–	–	–	ND	1.88 ± 0.06	*No match*	*-*

‘-’ negative**/**absent, **‘**+**’** mild positive**/**present, **‘**++**’** moderately positive, **‘**+++**’** strongly positive, **‘**+++*’ highly positive, SP- Siderophore production, PS-Phosphate solubilization, NF-Nitrogen Fixation, IAA- Indole Acetic Acid production (µg/ml), ND- Not Done, nifH^+^ **(**Fe subunit of nitrogenase gene). Bold values are the highest IAA production in greenhouse isolates.

**Table 2:** Bacterial characterization of the isolates from the rhizosphere of snowbrush ceanothus from the greenhouse conditions based on catalase production, siderophore, phosphate solubilization, nitrogen fixation, and IAA production.

S. No.	Code	Catalase	SP	PS	NF		IAA	BLAST	Accession numbers
					Media	*nif*H^+^			
1	GK_GR_41	++	+++	++	++	**+**	**14.08 ± 0.58**	*Pseudomonas* sp.	OP407589
2	GK_GR_42	++	+	+++	+++	–	6.05 ± 0.27	*Pseudomonas* sp.	OP407590
3	GK_GR_44	–	–	–	–	ND	1.49 ± 0.02	*Streptomyces* sp.	OP407592
4	GK_GR_45	–	+	+++	+++	–	7.35 ± 0.04	*Pseudomonas* sp.	OP407593
5	GK_GR_51	+	++	–	–	ND	1.69 ± 0.05	*Variovorax* sp.	OP407594
6	GK_GR_52	++	+++*	+++	+++	–	11.33 ± 1.23	*Pseudomonas* sp.	OP407595
7	GK_GR_55	++	+++*	++	+++	–	9.82 ± 0.17	*Pseudomonas* sp.	OP407596
8	GK_GR_58	+	–	–	–	ND	1.08 ± 0.04	*Streptomyces* sp.	OP407597
9	GK_GR_59	+	–	–	–	ND	2.19 ± 0.06	*Priestia* sp.	OP407598
10	GK_GR_60	+	+++*	+++	++	–	12.27 ± 0.04	*Pseudomonas* sp.	OP407599
11	GK_GR_61	+	–	–	–	ND	12.02 ± 0.40	*Agrobacterium* sp.	OP407600
12	GK_GR_64	+	+++	+	+++	+	10.60 ± 0.17	*Pseudomonas* sp.	OP407601
13	GK_GR_66	–	+++	–	++	+	3.82 ± 0.03	*Pseudomonas* sp.	OP407602
14	GK_GR_68	+	–	–	–	ND	5.27 ± 0.05	*Streptomyces* sp.	OP407603
15	GK_GR_70	++	+	–	–	ND	6.25 ± 0.43	*Xenophilus* sp.	OP407604
16	GK_GR_72	+	++	–	–	ND	6.65 ± 0.50	*Xenophilus* sp.	OP407605
17	GK_GR_73	+	++	+	+	–	3.46 ± 0.06	*Bacillus* sp.	OP407606
18	GK_GR_74	++	+	–	–	ND	3.09 ± 0.17	*Priestia* sp.	OP407607
19	GK_GR_75	–	–	–	–	ND	8.96 ± 0.33	*Priestia* sp.	OP407608
20	GK_GR_79	–	+	–	–	ND	7.00 ± 0.57	*Acidovorax* sp.	OP407609
21	GK_GR_81	–	+	–	–	ND	2.01 ± 0.09	*Pedobacter* sp.	OP407610
22	GK_GR_88	+	–	–	–	ND	3.18 ± 0.10	*Priestia* sp.	OP407611
23	GK_GR_90	+	+++*	++	++	+	5.19 ± 0.16	*Pseudomonas* sp.	OP407612
24	GK_GR_94	+	+	+++	++	–	4.16 ± 0.02	*No match*	*-*
25	GK_GR_97	++	++	–	–	ND	0.03 ± 0.05	*Peribacillus* ap.	OP407613
26	GK_GR_98	++	++	+	+++	+	11.79 ± 0.08	*Pseudomonas* sp.	OP407614
27	GK_GR_99	++	+	–	–	ND	2.39 ± 0.07	*Brevundimonas* sp.	OP407615
28	GK_GR_104	+	+++	+	+++	–	5.49 ± 0.09	*No match*	*-*
29	GK_GR_106	+	+	+	+	–	5.82 ± 0.23	*Ancylobacter* sp.	OP407616
30	GK_GR_109	–	+++	++	++	–	0.49 ± 0.19	*No match*.	*-*
31	GK_GR_111	+	–	–	–	ND	3.28 ± 0.07	*Streptomyces* sp.	OP407617
32	GK_GR_112	+	+++	–	+++	–	3.86 ± 0.06	*Pseudomonas* sp.	OP407618
33	GK_GR_115	+	+++*	–	+++	+	4.68 ± 0.06	*Pseudomonas* sp.	OP407619
34	GK_GR_119	+	++	+++	+++	+	0.43 ± 0.07	*Pseudomonas* sp.	OP407620
35	GK_GR_122	+	–	–	–	ND	6.29 ± 0.09	*Streptomyces* sp.	OP407621
36	GK_GR_124	++	–	–	–	ND	0.05 ± 0.03	*No match*	*-*

‘-’ negative/absent, ‘+’ mild positive/present, ‘++’ moderately positive, ‘+++’ strongly positive, ‘+++*’ highly positive, SP- Siderophore production, PS-Phosphate solubilization, NF-Nitrogen Fixation, IAA- Indole Acetic Acid production (µg/ml), ND- Not Done, nifH+(Fe subunit of nitrogenase gene). Bold values are the highest IAA production in greenhouse isolates.

### 2.6 Bacterial characterization

The bacterial colonies were isolated based on morphological features (Reynolds, 2018) and Gram stain using BD BBL™ Gram Stain Kits (BD Diagnostics) (**Figure S2A, Tables S1, S2**). The bacterial isolates were then tested for their ability to produce siderophores, indole acetic acid (IAA), and catalase, solubilize phosphate and fix atmospheric nitrogen. A single colony was picked and placed on a glass slide, and 1-2 drops of hydrogen peroxide were mixed. Bubbling shows a positive result for catalase activity (Pakpour and Horgan, 2021). The catalase test was repeated three times. The bacterial isolates were screened for phosphate solubilization on Pikovskaya medium three times (Pikovskaya, 1948) (HiMedia). The colonies were streaked onto this medium and incubated at 28°C for four days or until a clear halo was observed (Chung et al., 2005) (**Figure S2B**). The screening was repeated three times. *Bacillus megaterium ATCC14581* was used as a positive control. The siderophore-producing bacteria were screened on CAS (chrome azurol S) agar (Millipore SIGMA) (Schwyn and Neilands, 1987). The bacterial samples were streaked onto it and incubated at 28°C for four days or until a yellow-orange halo was observed on blue-colored media (**Figure S2C**) (Gamit and Tank, 2014). We used *Pseudomonas chlororaphis* O6 (produce pyoverdine siderophore) and positive control for the siderophore test. Nitrogen-fixing (NF) bacterial isolates were screened on Norris Glucose Nitrogen-Free Medium (NGNF) (HIMEDIA) for their ability to fix atmospheric nitrogen. The bacterial isolates were streaked on the plates and incubated at 28 °C for three days or until a clear zone around the colony appeared. The appearance of a clear zone indicates a positive result for nitrogen fixation (Wafula and Murunga, 2020; Gaete et al., 2022) (**Figure S2D**). *Rhizobium leguminosarum* C6 isolates used as a positive control.

The screening for NF bacterial isolates on NGNF Medium was done three times. The positive isolates for Nitrogen-fixing bacteria on NGNF Medium were amplified with PolF- (5′ TGC GAY CCS AAR GCB GAC TC 3′), PolR- (5′ ATS GCC ATC ATY TCR CCG GA 3′), universal primers for the presence of Nitrogenase gene (Poly et al., 2001). The bacterial genomic DNA was extracted from bacterial isolates (Thungapathra et al., 2002), and 50ng DNA was used to amplify 393bp fragment of Fe protein subunit of nitrogenase gene using Dream Taq polymerase in a 20µl reaction mix. The PCR was as follows: 2 min of initial denaturation at 95°C, followed by 30 cycles of 1min denaturation at 95°C, 1 min annealing at 55 °C, 2 min of extension at 72°C, then the final extension of 5 min 72 °C. The bacterial isolates were tested for IAA production by a colorimetric method described elsewhere (Sarker and Al-Rashid, 2013). The bacterial colonies were cultured in 5 ml LB broth (Fisher Scientific) supplemented with 0.1% tryptophan (EMD Millipore Corporation) at 28°C for 72 hours. A non-inoculated culture broth was used as a control. The supernatant was collected by centrifugation at 10000 rpm for 10 minutes. One ml of the Salkowski reagent (Salkowski’s reagent was prepared by mixing 2 ml of 0.5 mM FeCl_3_ in 49 ml of water and then carefully adding 49 ml of 70% perchloric acid (SIGMA-ALDRICH) was mixed with 1 ml of the supernatant and incubated for 25 minutes. IAA producing isolate developed pink color (**Figure S2F**) and read at 530 nm in the Spectramax Microplate reader (Molecular Devices). An IAA (SIGMA) standard curve was prepared with IAA (SIGMA) in the at 0, 5, 10, 20, 50, and 100 µg/ml of concentrations (**Figure S3**) and used to calculate the amount of IAA in each isolate (**Figure S2E**). The three replicates were taken for each bacterial isolate, and IAA content was calculated as per the equation obtained from IAA standard curve, and standard error was calculated.

### 2.7 Native soil characteristics

The fresh weight of 40 grams of bulk soil from each sample was taken and dried in a hot air oven at 60 °C for 72 hours, and dry weight was measured. Soil moisture percentage was calculated using the following equation


$$Soil\;moisture\;percentage=\frac{\left(wet\;soil\;weight\right)-\left(dry\;soil\;weight-bag\;weight\right)}{dry\;soil\;weight}\times 100$$

The pH and EC of the snowbrush ceanothus native soil were estimated by adding one part of the soil to two-part distilled water to make a soil slurry. A few drops of clear solutions from this slurry were taken to measure the pH and EC by an EC and pH meter (Thermo Scientific Orion Star A112 2011) (Cavins et al., 2008).

The total carbon (TC) and available phosphorus were measured in the bulk soil samples collected from the snowbrush ceanothus plants in Tony Grove. Triplicates were used per sample for each test. Five grams of each soil sample were air-dried for 72 hours. Dried samples were crushed using a mortar and pestle and sieved through a 250 µm sieve. Total carbon content was measured in dried soil samples of 60 to 230 mg using the SKALAR Carbon Analyzer 2008. The amount of phosphorus was calculated using Olsen’s sodium bicarbonate extraction method (Baxter, 2018). One gram of soil was added to 20ml of NaHCO_3_ (0.5 mol/L), and ammonium molybdate ascorbic acid was added. The available P was measured calorimetrically at 880 nm (SpectraMax M2, Molecular Devices, Sunnyvale, CA). A blank was used without soil. A standard curve for inorganic phosphate was plotted using KH_2_PO_4_ (potassium dihydrogen phosphate), and the amount of available phosphorus (in µg/g) was determined. An analysis of variance (ANOVA) and Tukey-Kramer method for multiplicity at α = 0.05 was done to depict the statistical differences among different samples on SAS Studio (SAS Institute, Cary, NC).

The available potassium and nitrogen were measured in the native and greenhouse soil samples (control and treatment) at the Utah State University Analytical Laboratories (USUAL) using Olsen NaHCO_3_ Method (Olsen et al., 1954) and the Nitrate-N analysis in Ca(OH)_2_ extract (Haby, 1989), respectively. The micronutrient test (Fe, Zn, Cu, Mn) for the same soil samples was also tested at the USUAL using the DTPA extractable elements technique (Lindsay and Norvell, 1978).

## 3 Results

### 3.1 Effect of native soil on the growth and development of snowbrush ceanothus plants

**Figure 1:**
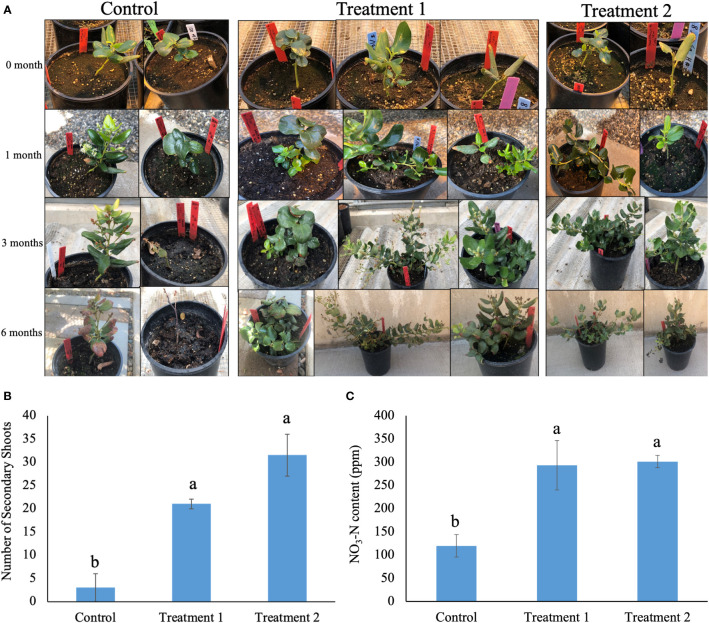
**(A)** Change in the growth of snowbrush ceanothus plants (with and without native soil) in six months. The plants treated in treatment 1 (native soil form location 1965m AMSL), and treatment 2 (native soil from location 2289m AMSL) showed bigger plants compared to control plants (not treated with native soil). The pot diameter is 16cm. **(B)** Significant increase in the number of secondary shoots of snowbrush ceanothus plants from cuttings treated with native soil (Treatment 1 and 2) compared to control plants after six months of treatment. **(C)** Significant increase in nitrate- nitrogen (NO_3_-N) content of snowbrush ceanothus plants after six months of treatment compared to control. The same letters denote no significant difference among treatments by Tukey’s method for multiplicity at α< 0.05.

The native soil-treated snowbrush ceanothus plants showed a visual difference in growth compared to the control plants (**Figure 1A**). The snowbrush ceanothus plants treated with native soil from both elevations showed a significant increase in secondary shoot numbers compared to non-treated plants (control) after six months of treatment (**Figure 1B**). The plants in treatments 1 and 2, treated with native soil from both elevations, showed a significant increase in nitrate-nitrogen content compared to the control (no native soil treatment) after six months (**Figure 1C**). The plant width and height were also measured after six month of treatment, but no significant difference observed between treatments (**Figure S1**).

### 3.2 The bacterial population in the rhizosphere of snowbrush ceanothus plants from the greenhouse

**Figure 2:**
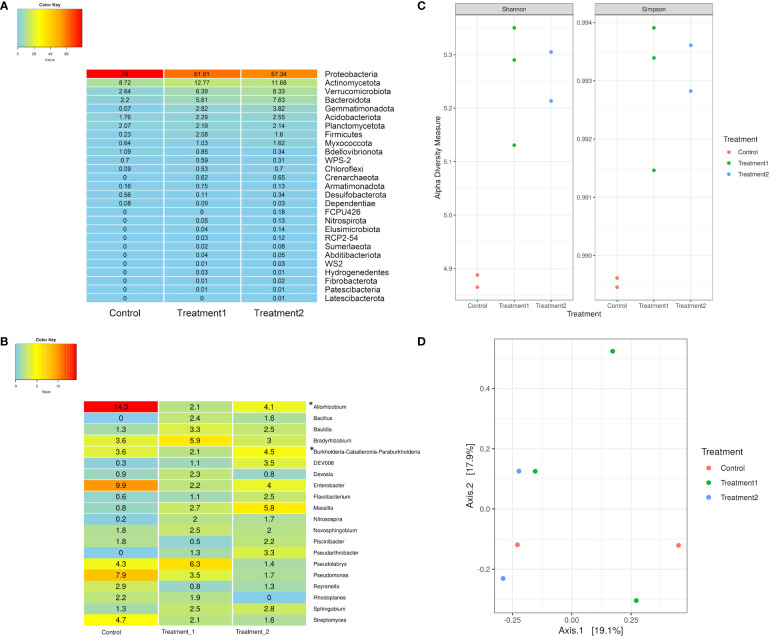
**(A)** Percent relative read abundance of control and treatment samples of snowbrush ceanothus from greenhouse conditions at the phyla level. **(B)** Percent relative read abundance of control and treatment samples at the genus level. **(C)** Alpha diversity (both Shannon and Simpson) revealed higher diversity in the treated samples when compared with the control samples. **(D)** Beta diversity of samples from the greenhouse showed a similar type of microbial composition within all the rhizosphere samples.*Allorhizobium- Allorhizobium, Neorhizobium, Pararhizobium, and *Rhizobium*. *Burkholderia- Burkholderia, Caballeronia, and *Paraburkholderia*.

The 16S rRNA sequencing data were analyzed to study the microbial populations in the rhizosphere samples of control and treated plants. Taxonomic classification at the phyla level revealed the dominance of the phyla *Actinomycetota*, *Bacteroidota*, and *Verrucomicrobiota* in the treated rhizosphere samples compared to the control samples (**Figure 2A**). Genus-level classification revealed the relative read abundance (percent) of *Allorhizobium-Neorhizobium-Pararhizobium-Rhizobium, Massilia, Nitrosospira, Bradyrhizobium*, and *Sphingobium* in both treated and control samples (**Figure 2B**). It also revealed the presence of *Bacillus* and *Pseudoarthrobacter* in the treatment samples but their absence in control (**Figure 2B**). Alpha and beta diversity are higher-order measures to describe microbiome samples that depict broader differences in the composition of microbes. Alpha diversity describes within sample diversity and beta diversity measures between sample diversity. Alpha diversity measures (both Shannon and Simpson) revealed a higher diversity in the treated samples when compared with the control samples (**Figure 2C**). Higher within-sample-diversity of treated samples indicates colonization of plant’s rhizosphere region by larger number of bacterial strains than control due to treatment with native soil. Beta diversity analysis did not show any distinct grouping of samples. Despite the different relative abundance, they showed a similar type of microbial composition within all the rhizosphere samples (**Figure 2D**).

### 3.3 Isolation and identification of PGPR

Sixty-nine bacterial colonies were isolated from the rhizosphere collected from the native region. Nine colonies were isolated from Minimal M9 Media (MM9), 22 from ¼ Tryptic Soy Agar (TSA), 21 from ¼ Nutrient Agar (NA), six from Actinomycete Isolation Agar (AIA), and 11 were from Yeast Mannitol Agar (YMA) (**Table S1**). Twenty-seven out of the 69 colonies were selected for 16S rRNA sequencing, followed by a BLAST search for identification. The bacterial isolates were characterized based on the colonies’ color, texture, transparency, size, consistency, and morphology (**Tables S1**, **2**). Of these 27, nine were from ¼ TSA, 12 from ¼ NA, two from MM9, one from AIA, and three from YMA (**Table S1**). The BLAST results of the 27 colonies from the native soil revealed the presence of bacterial species from the genus *Streptomyces, Nocardia, Neorhizobium, Pseudomonas, Xenophilus, Promicromonospora, Pedobacter*, and *Pantoea* are amongst a few others. *Streptomyces* was the most abundant genus, as 44% of the identified bacterial samples belonged to it (**Table 1**).

Seventy-eight bacterial colonies were isolated from the rhizosphere of the snowbrush ceanothus grown in greenhouse conditions. The variable region of 16S rRNA was sequenced and identified in 36 purified bacterial colonies against the 16S rRNA database on NCBI. Out of all the bacterial colonies isolated, 13 colonies were from MM9 media, 33 from ¼ NA, 14 from ¼ TSA, nine from AIA, and nine from YMA (**Table S2**). Out of 36 colonies, eight were from MM9 media, 14 from ¼ NA, four from AIA, six from ¼ TSA, and four from YMA media. BLAST search revealed the presence of *Streptomyces, Pseudomonas, Variovorax, Priestia, Bacillus, Xenophilus, Acidovorax, Ancylobacter*, and *Pedobacter*. Most isolates belonged to *Pseudomonas* (41.7%) and *Streptomyces* (19%) (**Table 2**).

### 3.4 Native soil characteristics

The native soil from 1950m has significantly more carbon and manganese content than the native soil from 2289m. The native soil from location 1950m has a salinity of 0.2 µS/cm and is significantly higher than location 2289, which is 0.1 µS/cm. The other components in the native soil from both elevations are the same (**Table 3**).

**Table 3:** Native soil characteristics from both elevations.

Sample	Carbon (%)	Nitrate nitrogen (mg/kg)	Phosphorus (µg/g)	Potassium (mg/kg)	Zinc (mg/kg)	Iron (mg/kg)	Copper (mg/kg)	Manganese (mg/kg)	Soil Moisture (%)	pH	EC (µS/cm)
**1950**	24.8a	16.9a	6.0a	600.5a	2.2a	83.9a	0.8a	26.7a	30.2a	6.2a	0.2a
**2289**	12.8b	1.9a	34.8a	485.5a	2.6a	74.1a	1.0a	18.5b	32.1a	6.4a	0.1b

Same letters within a column denote no significance change among different samples by Tukey’s method for multiplicity at α = 0.05.

### 3.5 Bacterial characterization

The bacterial isolates were morphologically characterized based on the colonies’ color, texture, transparency, size, consistency, and morphology (**Tables S1, S2**) (**Figure S2A**). The Gram stain results revealed that 74% of the isolates from native soil were Gram-positive, and 26% of them turned out to be Gram-negative (**Table S1**). The greenhouse snowbrush ceanothus rhizosphere extracted from the treated plants had 23 Gram-negative bacteria (64%) and 13 Gram-positive bacteria (36%) (**Table S2**).

#### 3.5.1 Bacterial isolates tested for catalase production

**Figure 3:**
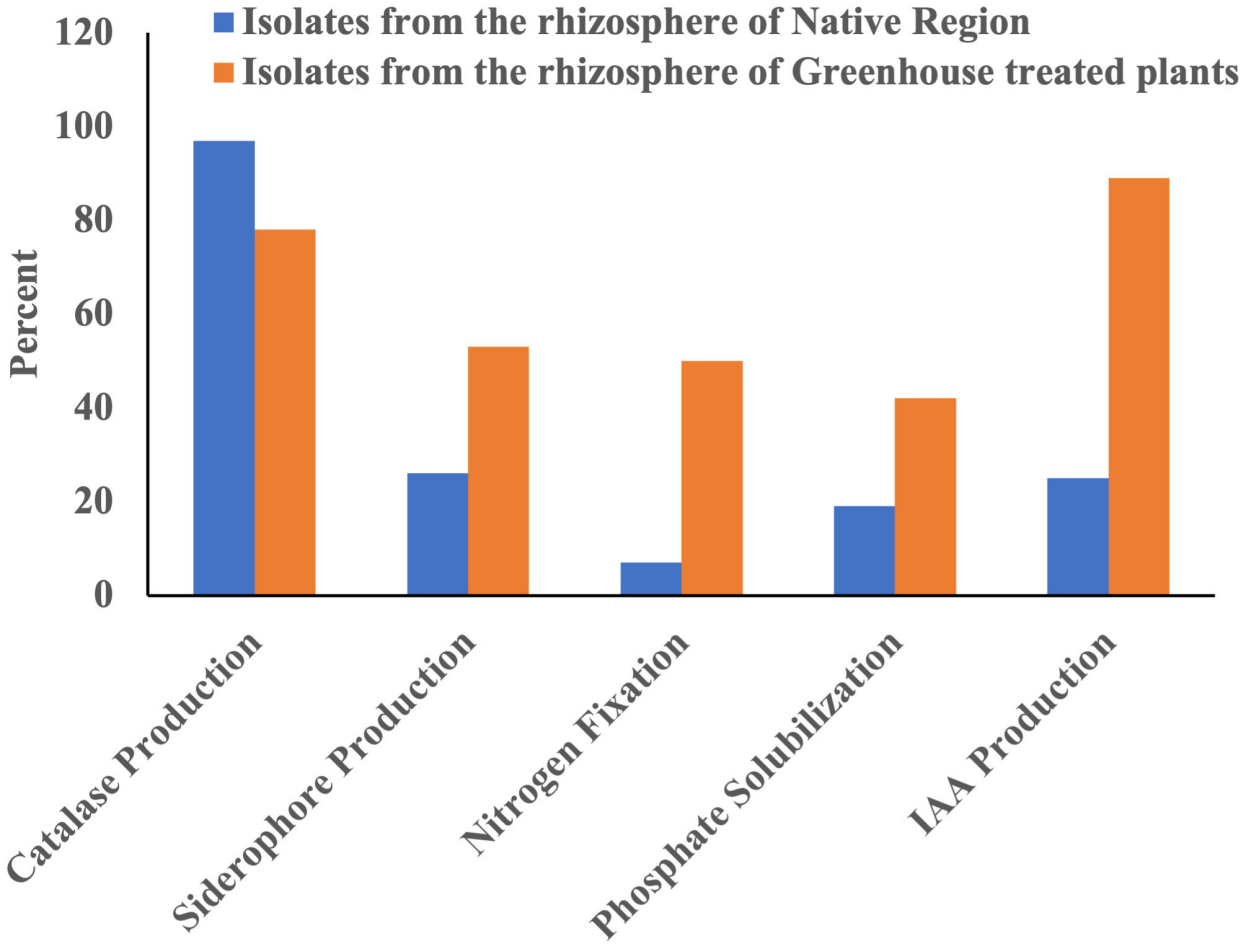
The abundance of isolates for plant growth-promoting traits in the rhizosphere of snowbrush ceanothus from native region and the rhizosphere of greenhouse treated snowbrush ceanothus plants.

Ninety-seven percent of the total isolates from native rhizosphere soil were catalase-positive (**Tables 1** and **Figure 3**). In the greenhouse isolates from the treated plants, 78% were catalase-positive (**Table 2** and **Figure 3**).

#### 3.5.2 Bacterial isolates tested positive for Phosphate solubilization on Pikovskaya medium

Five of the 27 bacterial isolates from the native soil showed a clear halo around the colony, indicating a 19% positive result for phosphate solubilization (**Figure S2B**) (**Table 1** and **Figure 3**). Fifteen isolates showed clear halo from the snowbrush ceanothus rhizosphere in the greenhouse (**Table 2**). About 42% of the total isolates from the snowbrush ceanothus rhizosphere in the greenhouse showed phosphate solubilization (**Figure 3**). The majority of isolates belonged to the genus *Pseudomonas*. Some other genera included *Streptomyces, Bacillus, Ancylobacter*, and *Pantoea*.

#### 3.5.3 Bacterial isolates tested positive for Siderophore production on CAS (Chrome Azurol S) agar

Seven of 27 bacterial isolates from the native soil showed a yellow-orange halo around the bacterial colony, indicating that 26% of the bacterial isolates can produce siderophores (**Figure S2C**) (**Table 1** and **Figure 3**). Nineteen out of 36 (53%) bacterial isolates from the greenhouse snowbrush ceanothus rhizosphere showed siderophore production (**Table 2** and **Figure 3**). Five isolates showed a bigger and brighter halo than the others (**Table 2**). Most of the isolates that showed siderophore production belonged to the genus *Pseudomonas*. Some others included *Streptomyces, Variovorax, Xenophilus*, *Peribacillus, Priestia, Pedobacter, Brevibacterium, Leifsonia*, and *Bacillus*.

#### 3.5.4 Bacterial isolates tested positive for Nitrogen fixation based on Norris Glucose Nitrogen free agar media and the presence of the *nif*H^+^ gene

Eighteen bacterial isolates (50%) from the greenhouse-treated plants’ rhizosphere showed nitrogen fixation ability using Norris Glucose Nitrogen Free media but 7 of them amplified the 393bp *nif*H^+^ gene (Fe subunit of nitrogenase gene) (**Table 2** and **Figure 3**). Two of the bacterial isolated from the rhizosphere of snowbrush ceanothus from the native soil revealed a clear halo around them (**Figure S2D**), indicating that 7% of them can fix atmospheric nitrogen, and none of them amplified the 393bp *nifH^+^* gene (**Table 1** and **Figure 3**). Most of this study’s isolates that tested positive for nitrogen fixation belonged to *Pseudomonas.* Others were *Bacillus*, *Ancylobacter*, and *Pantoea*.

#### 3.5.5 Bacterial isolates tested positive for IAA production

The IAA test results in the bacterial isolates from the rhizosphere of native soil samples revealed that seven out of the 27 isolates (26%) produced more than 1 µg/ml of IAA (**Table 1** and **Figure 3**). IAA production observed in the samples ranged from approximately 1 to 33 µg/ml (**Tables 1**, **2**). These IAA- producing isolates belonged to *Pantoea, Brevibacterium, Leifsonia*, and *Streptomyces*. Two of them, GK_NR_133 (*Pantoea* sp.) and GK_NR_149 (*Brevibacterium* sp), produced the highest amounts of IAA, 33.52 ± 0.15 µg/ml and 14.88 ± 0.11 µg/ml, respectively (**Table 1**).

Out of all the bacteria isolated from the greenhouse snowbrush ceanothus rhizosphere, 32 out of 36 or 89% of the isolates from snowbrush ceanothus treated plants produced more than 1 µg/ml of IAA (**Figure 3**). Six bacterial isolates produced more IAA ranging from 11-14 µg/ml (**Table 2**). They belonged to the genera *Pseudomonas, Agrobacterium, Priestia, Acidovorax, Xenophilus, Streptomyces, Ancylobacter, Bacillus*, and *Brevundimonas.*

Thirteen of the 27 identified bacterial isolates showed two or more PGP traits from the native soil samples, while one bacterial isolate showed all five PGP traits (**Tables 1**, **4**). Twelve of 36 bacterial isolates from the greenhouse snowbrush ceanothus rhizosphere showed a positive result for all the PGP traits. Besides, 34 isolated bacteria from the greenhouse snowbrush ceanothus rhizosphere showed two or more PGP traits (**Table 2**). Among all the bacteria isolated, 13 isolates tested positive for all the PGP traits evaluated and belonged to *Pseudomonas, Pantoea, Bacillus*, and *Ancylobacter* (**Table 4**). One of these isolates GK_NR_133 was from the rhizosphere of snowbrush ceanothus from the native region, and the other twelve isolates, GK_GR_41, GK_GR_42, GK_GR_52, GK_GR_55, GK_GR_60, GK_GR_64, GK_GR_73, GK_GR_90, GK_GR_94, GK_GR_98, GK_GR_104, and GK_GR_106 were from the rhizosphere of snowbrush ceanothus treated with the native soil under greenhouse conditions.

**Table 4:** List of bacterial isolates that tested positive for all the PGP traits.

S.No.	Code	Catalase	SP	PS	NF		IAA	BLAST	Accession numbers
					Media	*nifH*			
1	GK_NR_133	+	+	+++	+++*	–	33.52 ± 0.15	*Pantoea* sp.	OP407625
2	GK_GR_41	++	+++	++	++	+	14.08 ± 0.58	*Pseudomonas* sp.	OP407589
3	GK_GR_42	++	+	+++	+++	–	6.05 ± 0.27	*Pseudomonas* sp.	OP407590
4	GK_GR_55	++	+++*	++	+++	–	9.82 ± 0.17	*Pseudomonas* sp.	OP407596
5	GK_GR_52	++	+++*	+++	+++	–	11.33 ± 1.23	*Pseudomonas* sp.	OP407595
6	GK_GR_60	+	+++*	+++	++	–	12.27 ± 0.04	*Pseudomonas* sp.	OP407599
7	GK_GR_64	+	+++	+	+++	+	10.60 ± 0.17	*Pseudomonas* sp.	OP407601
8	GK_GR_73	+	++	+	+	–	3.46 ± 0.06	*Bacillus* sp.	OP407606
9	GK_GR_90	+	+++*	++	++	+	5.19 ± 0.16	*Pseudomonas* sp.	OP407612
10	GK_GR_94	+	+	+++	++	–	4.16 ± 0.02	No match	*-*
11	GK_GR_98	++	++	+	+++	+	11.79 ± 0.08	*Pseudomonas* sp.	OP407614
12	GK_GR_104	+	+++	+	+++	–	5.49 ± 0.09	No match	–
13	GK_GR_106	+	+	+	+	–	5.82 ± 0.23	*Ancylobacter* sp.	OP407616

‘-’ negative/absent, ‘+’ mild positive/present ‘++’ moderately positive, ‘+++’ strongly positive, ‘+++*’ highly positive, SP, Siderophore production; PS, Phosphate solubilization; NF, Nitrogen Fixation; IAA, Indole Acetic Acid production (µg/ml); ND, Not Done; nifH+ (Fe subunit of nitrogenase gene).

## 4 Discussion

Various studies show that rhizosphere microbes help in the growth of plants in different ways, such as promoting growth and increasing biomass by secreting volatile compounds and growth inducers and inducing resistance to biotic and abiotic stresses (Ryu et al., 2004; Van Der Heijden et al., 2008; Lugtenberg and Kamilova, 2009). The diversity and richness of rhizosphere microbes enhance the productivity of plants aboveground under different environmental conditions (Wagg et al., 2011). Our results suggest that the greenhouse-grown snowbrush ceanothus plants treated with native soil showed improved growth of plants compared to control. The treated plants showed a significant increase in the number of secondary shoots and nitrate-nitrogen contents in their soil (**Figures 1A–C**). Snowbrush ceanothus is an evergreen shrub that expands in width by forming secondary shoots, which is why the number of secondary shoots is the growth parameter we choose in this study. Because of the limited sample size, the growth parameters such as plant width and height are not statistically significant (**Figure S1**). Nitrate - nitrogen content of the soil is a good indicator of available nitrogen to the plant (Guo et al., 2014). The significant increase in the number of secondary shoots and nitrate content in native soil treated greenhouse-grown plants indicated that the microbes present in the native habitat of the Snowbrush ceanothus have some positive impact on growth and development of these plants in the greenhouse.

The metagenomic analysis of the rhizosphere of the native soil treated and non-treated (control) plants revealed the significant dominance of the bacteria belonging to phyla *Actinomycetota*, *Bacteroidota*, and *Verrucomicrobiota* in the treated samples compared to the control (**Figure 2A**). However, the abundance of bacteria belonging to the phylum *Proteobacteria* is almost similar in all the samples. These are common phyla found in the rhizosphere soil of other crop plants, such as maize and sugarcane (Pisa et al., 2011; Correa-Galeote et al., 2016). Many phyla in the treated greenhouse snowbrush ceanothus rhizosphere showed percent read abundance to be zero in control rhizosphere samples. These findings also indicate the presence of plant growth-promoting microbes in the native soil, which is lacking in the greenhouse soil. These results suggest that the microbes present in the natural habitat of snowbrush ceanothus are essential for its growth and development and resilience to its native conditions.

We found that most of the bacterial isolates in our study belong to the *Pseudomonas* and *Streptomyces*. In this study, bacteria from genus *Streptomyces* (41%) dominated the isolates from the rhizosphere of the natural habitat (**Table 1**). However, 19% of the isolates from greenhouse-grown treated plants belong to the genus *Streptomyces* (**Table 2**). *Streptomyces* is a genus from phyla *Actinomycetota* and is known to have several bacterial species which possess PGP traits: *Streptomyces longisporoflavus* can solubilize phosphate (Nandimath et al., 2017); *Streptomyces umbrinus*, a halotolerant bacteria that can produce siderophores (Etesami and Glick, 2020).

Most bacterial isolates from the rhizosphere of greenhouse-grown treated plants belong to the genus *Pseudomonas* (**Tables 1** and **2**). Eight out of 13 shortlisted isolates tested positive for all PGP traits belonging to the genus *Pseudomonas*, and all of them were isolated from the rhizosphere of greenhouse-grown snowbrush ceanothus plants treated with native soil (**Table 4**). Many species in this genus carry out plant growth-promoting functions, such as *Pseudomonas koreensis*, which can execute nitrogen fixation in sugarcane (Li et al., 2017) and act as a biocontrol agent (Gu et al., 2020). *Pseudomonas chlororaphis* can act as a biocontrol agent, is salt-tolerant, and solubilizes zinc (Egamberdieva, 2012; Shahid et al., 2017).

The isolate GK_GR_73 from the greenhouse-treated rhizosphere was identified as *Bacillus* and tested positive for all four PGP traits (**Table 4**). In the metagenomic study, the bacteria belonging to the genera *Bacillus* and *Pseudoarthrobacter* were present in the native soil treated snowbrush ceanothus rhizosphere but absent in control (**Figure 2B**). *Bacillus* is a well-known genus containing PGPR that produces siderophores, fixes nitrogen, is a biocontrol agent, and can help plants in biotic and abiotic stresses.

Four isolates identified in the genus *Priestia*, GK_GR_74, tested positive for siderophore production and IAA, whereas GK_GR_59, GK_GR_75, and GK_GR_88 tested positive for IAA production (**Table 2**). The genus *Priestia* belonged to the phyla *Bacillota* (*Firmicutes*) reported to have various species of the *Priestia* possess plant growth-promoting activities and help plants grow and develop. *Priestia aryabhattai* promotes plant growth in soybean by increasing phytohormones, such as ABA and GA, and is also tolerant to oxidative stress (Park et al., 2017). Bacterium *Priestia megaterium* promotes growth in mustard by phosphate solubilization and capsicum by zinc solubilization (Kang et al., 2014; Bhatt and Maheshwari, 2020).

The bacterial isolates of *Xenophilus* are reported to play a role in bioremediation as the aerobic Azoreductase enzyme from strain *Xenophilus azovorans* KF46F has been cloned and has a significant potential to use as an aerobic treatment to degrade Azo dye contamination in wastewater plants (Blümel et al., 2002). One isolate, GK_NR_144, identified as *Xenophilus* sp., tested positive for siderophore production, and two isolates, GK_GR_70 and 72, also identified as *Xenophilus* sp., tested positive for IAA along with siderophore production (**Table 1** and **2**). Further characterization of the isolates from our study for their role in bioremediation is needed.

Studies have shown that the genus *Pantoea* can fix nitrogen (Suleimanova et al., 2021) in free-living and symbiotic states (Nadarasah and Stavrinides, 2014). Many species from the genus *Pantoea* have been shown to produce IAA and promote plant growth, such as *Pantoea ananatis* has been shown to solubilize phosphate and potentially promote plant growth in the rhizosphere (Bakhshandeh et al., 2014; da Silva et al., 2015). The first few members of *Pantoea* were known as plant pathogens, but later many *Pantoea* strains were isolated from the various aquatic and terrestrial environments, including humans (Walterson and Stavrinides, 2015). One isolate, GK_NR_133, was identified in the genus *Pantoea.* This isolate tested positive for all the plant growth-promoting traits tested in the study (**Table 4**). The highest IAA production was observed by the isolates from native soil, which belonged to the genera *Pantoea* (33.52 ± 0.15 µg/ml) and *Brevibacterium* (14.88 ± 0.11 µg/ml) (**Table 1**). *Pantoea* is previously known to be high IAA-producing genera (Apine and Jadhav, 2011). High levels of IAA are known to promote the formation of lateral roots and increase the primary root length and surface area (Gupta and Pandey, 2019). The presence of species related to the *Pantoea* genus in the native soil of snowbrush ceanothus can be one of the reasons for the high drought tolerance of this plant.

Snowbrush ceanothus is an evergreen plant that survives well in a dry arid climate, is drought and heat-tolerant, and fixes nitrogen. It is an ornamental plant for low-water-use landscaping and an excellent resource for plant growth-promoting microbes. The bacteria isolated from this plant showed characteristics to fix nitrogen, to produce siderophore and IAA, and solubilize phosphate. Hence this study provided a list of the isolates that have the potential as an inoculant in bio-fertilizer and bio-stimulant in sustainable organic agriculture. Two bacterial isolates GK_GR_94 and GK_GR_104, did not show a match with any identified bacterium against the 16S rRNA database, one belonging to the genus *Pantoea*, eight *Pseudomonas* isolates, one *Bacillus* sp., and one *Ancylobacter* sp., that tested positive for all the plant growth-promoting activities. Besides, there are isolates belonging to the genus *Streptomyces*, *Bacillus*, *Peribacillus*, *Variovorax*, *Xenophilus*, *Brevibacterium*, and *Priestia*, which exhibit at least one of the plant growth-promoting activities tested in the study. These isolates are being tested on model plants *Arabidopsis thaliana* and *Medicago truncatula* for plant growth activities in greenhouses. In the future, these will be tested on crops such as corn, wheat, and alfalfa in greenhouses and fields and precisely identified by Whole-Genome Sequencing.

## Data availability statement

The data presented in the study are deposited in the BioProject: PRJNA853068 at NCBI repository, BioSample accessions SAMN29359218, SAMN29359219, SAMN29359220, SAMN29359221, SAMN29359222, SAMN29359223, SAMN29359224 and available at (http://www.ncbi.nlm.nih.gov/bioproject/853068).

## Author contributions

AK conceptualized the idea and designed it and supervised the experiments. JG isolated DNA and prepared the 16S rRNA library, isolated, identified, and characterized bacteria, and carried out the plant growth assays. VS carried out the metagenomic analysis. KH assisted JG with experiments. AK and KH isolated bacterial DNA and amplified *nif*H^+^ gene. AK, JG, and VS analyzed and interpreted the data. The manuscript was written jointly with contributions from all the authors. All authors have read and approved the manuscript.
