# Supplementary material for: Exploration of the rhizosphere microbiome of native plant Ceanothus velutinus – an excellent resource of plant growth-promoting bacteria
Source: Front Plant Sci. 2022 Dec 15;13:979069. doi: 10.3389/fpls.2022.979069 (PMC9798410; doi:10.3389/fpls.2022.979069)
Supplement: Supplementary file 1 [file DataSheet_1.docx]

Supplementary Material

# Supplementary Data

The metagenomic reads obtained by Illumina for 16srRNA in this study can be found in at <http://www.ncbi.nlm.nih.gov/bioproject/853068> BioProject ID: PRJNA853068

**
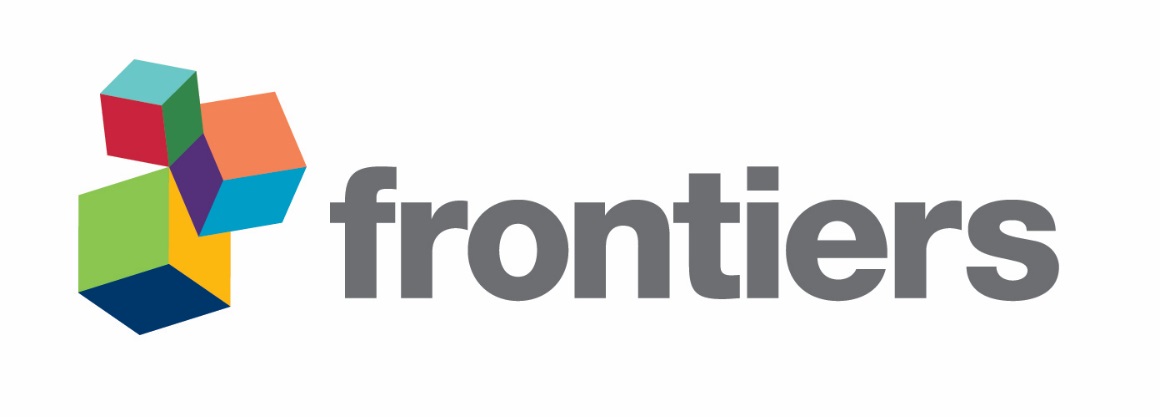
**

Table S 1 Bacterial isolation from the rhizosphere of snowbrush ceanothus from the native region and morphological characterization

| **S. No.** | **Code** | **Media** | **Color** | **Texture** | **Transparency** | **Size** | **Consistency** | **Morphology** | **Gram Stain** | **Catalase** | **BLAST** |
| --- | --- | --- | --- | --- | --- | --- | --- | --- | --- | --- | --- |
| 1 | GK_NR_127 | M9 | White (Bullseye) | Matte | Opaque | Dot | Dry | Raised | + | + | No match |
| 2 | GK_NR_188 | M9 | Yellow | Glossy | Translucent | Normal | Viscous | Flat | + | + | *Janthinobacterium* sp. |
| 3 | GK_NR_129 | 1/4 TSA | White/ Brown (Bullseye) | Matte | Opaque | Dot | Dry | Raised | + | + | *Streptomyces* sp. |
| 4 | GK_NR_130 | 1/4 TSA | Cream | 50/50 Glossy Matte | Translucent | Normal | Viscous | Normal | - | - | *Peribacillus* sp. |
| 5 | GK_NR_131 | 1/4 TSA | Cream/ Pink | Glossy | Translucent | Widespread | Mucus | Normal | - | + | *Neorhizobium* sp. |
| 6 | GK_NR_133 | 1/4 TSA | Yellow | Glossy | Translucent | Widespread | Viscous | Raised | + | + | *Pantoea* sp. |
| 7 | GK_NR_136 | 1/4 TSA | Off-White/ Brown (bullseye) | Matte | Opaque | Dot | Dry | Normal | - | ++ | *Streptomyces* sp. |
| 8 | GK_NR_139 | 1/4 TSA | White | Matte | Opaque | Dot | Dry | Chalky | + | + | *Nocardia* sp. |
| 9 | GK_NR_177 | 1/4 TSA | Yellow (ish)/ White | Glossy | Opaque | Dot | Viscous | Flat | - | + | *Promicromonospora* sp. |
| 10 | GK_NR_195 | 1/4 TSA | Yellow | 50/50 Glossy Matte | Translucent | Normal | Viscous | Cloudy | + | + | No match |
| 11 | GK_NR_196 | 1/4 TSA | Pink (ish) | Glossy | Translucent | Normal | Viscous | Flat | + | + | *Pedobacter* sp. |
| 12 | GK_NR_143 | 1/4 NA | Brown (dark) (Bullseye) | Matte | Opaque | Dot | Dry | Raised | + | + | *Streptomyces* sp. |
| 13 | GK_NR_144 | 1/4 NA | White (bullseye) | Matte | Opaque | Dot | Dry | Chalky | + | + | *Xenophilus* sp. |
| 14 | GK_NR_145 | 1/4 NA | Yellow/ White | Matte | Opaque | Dot | Dry | Raised | + | + | *Streptomyces* sp. |
| 15 | GK_NR_146 | 1/4 NA | Yellow (Bullseye) | Matte | Opaque | Dot | Dry | Normal | + | ++ | No match |
| 16 | GK_NR_149 | 1/4 NA | White | 50/50 Glossy Matte | Opaque | Dot | Viscous | Flat | + | + | *Brevibacterium* sp. |
| 17 | GK_NR_150 | 1/4 NA | Clear | Glossy | Transparent | Spread | Viscous | Flat | + | ++ | *Leifsonia* sp. |
| 18 | GK_NR_154 | 1/4 NA | Yellow (ish) | Glossy | Translucent | Spread | Viscous | Flat | + | ++ | *Agromyces* sp. |
| 19 | GK_NR_179 | 1/4 NA | White/ Dark (bullseye) | Matte | Opaque | Dot | Dry | Chalky, raised | + | + | *Streptomyces* sp. |
| 20 | GK_NR_180 | 1/4 NA | Brown (bullseye) | Matte | Opaque | Dot | Dry | Normal | + | + | *Streptomyces* sp. |
| 21 | GK_NR_182 | 1/4 NA | White (bullseye)/ Purple (center) | Matte | Translucent | Dot | Dry | Flat | + | + | *Streptomyces* sp. |
| 22 | GK_NR_194 | 1/4 NA | Clear | Matte | Transparent | Dot | Dry | Flat | + | + | *Pseudomonas* sp. |
| 23 | GK_NR_197 | 1/4 NA | Indigo/ Purple (deep) | 50/50 Glossy Matte | Translucent | Dot | Dry | Flat | - | + | No match |
| 24 | GK_NR_156 | AIA | Yellow (pale) | Matte | Translucent | Dot | Dry | Normal | - | + | *Staphylococcus* sp. |
| 25 | GK_NR_162 | YMA | Off-White/ Mustard/ Red (bullseye) | Matte | Opaque | Dot | Dry | Normal | - | ++ | *Streptomyces* sp. |
| 26 | GK_NR_166 | YMA | White | Matte | Opaque | Dot | Dry | Raised | - | + | *Streptomyces* sp. |
| 27 | GK_NR_186 | YMA | Off-White/ Yellow (center) (dark) | Matte | Opaque | Dot | Dry | Raised | - | + | *Streptomyces* sp. |

‘-’ negative, ‘+’ mild positive, ‘++’ moderately positive

Table S2. Bacterial isolation from the rhizosphere of snowbrush ceanothus under greenhouse conditions and morphological characterization

| **S. No.** | **Code** | **Media** | **Color** | **Texture** | **Transparency** | **Size** | **Consistency** | **Morphology** | **Gram stain** | **Catalase production** | **BLAST** |
| --- | --- | --- | --- | --- | --- | --- | --- | --- | --- | --- | --- |
| 1 | GK_GR_41 | M9 | Off-white | Glossy | Transparent | Normal | Mucus | Normal | - | ++ | *Pseudomonas* sp. |
| 2 | GK_GR_58 | M9 | White/ Dark brown (bullseye) | Matte | Opaque | Normal | Dry | Normal | - | ++ | *Streptomyces* sp. |
| 3 | GK_GR_59 | M9 | Tan (light) | 50/50 Glossy matte | Translucent | Spread | Dry | Wrinkly | + | - | *Priestia* sp. |
| 4 | GK_GR_74 | M9 | Mustard (dull) | 50/50 Glossy Matte | Translucent | Normal | Viscous | Small- matte/Big-Glossy | + | - | *Priestia* sp. |
| 5 | GK_GR_75 | M9 | White (off)/ Yellow | Matte | Opaque | Dot | Dry | Normal | - | - | *Priestia* sp. |
| 6 | GK_GR_94 | M9 | Tan/ White | Glossy | Translucent | Spread | Viscous | Normal | - | + | No Match |
| 7 | GK_GR_106 | M9 | White | Glossy | Translucent | Spread | Viscous | Normal | - | ++ | *Ancylobacter* sp. |
| 8 | GK_GR_109 | M9 | Off yellow | Glossy | Translucent | Spread | Viscous | Normal | - | ++ | No Match |
| 9 | GK_GR_55 | 1/4 TSA | White (off) | Glossy | Translucent | Normal | Mucus | Normal | + | + | *Pseudomonas* sp. |
| 10 | GK_GR_88 | 1/4 TSA | White | Matte | Translucent | Spread | Dry | Normal | + | + | *Priestia* sp. |
| 11 | GK_GR_90 | 1/4 TSA | Yellow (pastel) | Glossy | Opaque | Normal | Viscous | Normal | - | + | *Pseudomonas* sp. |
| 12 | GK_GR_119 | 1/4 TSA | Off-white | Glossy | Translucent | Spread | Viscous | Normal | - | + | *Pseudomonas* sp. |
| 13 | GK_GR_122 | 1/4 TSA | Yellow (pale) | Matte | Opaque | Dot | Dry | Raised | - | + | *Streptomyces* sp. |
| 14 | GK_GR_124 | 1/4 TSA | White | Glossy | Translucent | Normal | Viscous | Normal | - | - | No Match |
| 15 | GK_GR_42 | 1/4 NA | White | Glossy | Translucent | Normal | Viscous | Normal | + | + | *Pseudomonas* sp. |
| 16 | GK_GR_44 | 1/4 NA | Yellow/ Cream (bullseye) | Matte | Opaque | Dot | Dry | Raised | - | + | *Streptomyces* sp. |
| 17 | GK_GR_45 | 1/4 NA | Off-white/ Tan | Glossy | Translucent | Spread | Mucus | Normal | - | + | *Pseudomonas* sp. |
| 18 | GK_GR_60 | 1/4 NA | Tan | Glossy | Translucent | Normal | Viscous | Normal | + | ++ | *Pseudomonas* sp. |
| 19 | GK_GR_61 | 1/4 NA | White, Grey (bullseye) | Glossy | Translucent | Normal | Viscous | Normal | + | - | *Agrobacterium* sp. |
| 20 | GK_GR_64 | 1/4 NA | Yellow (light)/ White | Glossy | Translucent | Normal | Viscous | Normal | - | - | *Pseudomonas* sp. |
| 21 | GK_GR_79 | 1/4 NA | Clear | Matte | Transparent | Normal | Dry | Foggy | - | - | *Acidovorax* sp. |
| 22 | GK_GR_81 | 1/4 NA | Orange (pale)/ Pink | Glossy | Translucent | Normal | Viscous | Normal | + | + | *Pedobacter* sp. |
| 23 | GK_GR_97 | 1/4 NA | White/ Tan | Glossy | Translucent | Normal | Viscous | Normal | - | + | *Peribacillus* ap. |
| 24 | GK_GR_98 | 1/4 NA | White (tinge) | Glossy | Translucent | Widespread | Mucus | Normal | - | + | *Pseudomonas* sp. |
| 25 | GK_GR_99 | 1/4 NA | Orange (bright) | 50/50 Glossy Matte | Translucent | Dot | Dry | Normal | + | ++ | *Brevundimonas* sp. |
| 26 | GK_GR_111 | 1/4 NA | Tan/ Mustard | Matte | Opaque | Dot | Dry | Normal | - | ++ | *Streptomyces* sp. |
| 27 | GK_GR_112 | 1/4 NA | White, Tan, Pink (bullseye) | Glossy | Translucent | Spread | Viscous | Normal | - | ++ | *Pseudomonas* sp. |
| 28 | GK_GR_51 | AIA | Yellow | Matte | Transparent | Dot | Dry | Normal | - | + | *Variovorax* sp. |
| 29 | GK_GR_52 | AIA | White (off) | Glossy | Transparent | Normal | Mucus | Normal | - | + | *Pseudomonas* sp. |
| 30 | GK_GR_66 | AIA | Yellow/ White | Glossy | Opaque | Normal | Viscous | Normal | - | - | *Pseudomonas* sp. |
| 31 | GK_GR_68 | AIA | Orange/ Yellow | Matte | Opaque | Spread | Dry | Secretes yellow/orange pigment | + | + | *Streptomyces* sp. |
| 32 | GK_GR_115 | AIA | Yellow/ White | Glossy | Opaque | Normal | Viscous | Fuzzy | - | + | *Pseudomonas* sp. |
| 33 | GK_GR_70 | YMA | Yellow (mustard) | Matte | Translucent | Dot | Dry | Secretes mustard yellow pigment | - | + | *Xenophilus* sp. |
| 34 | GK_GR_72 | YMA | Yellow (light)/ White | Glossy | Transparent | Spread | Viscous | Normal | - | + | *Xenophilus* sp. |
| 35 | GK_GR_73 | YMA | White/ Yellow | 50/50 Glossy Matte | Opaque | Normal | Viscous | Blooming effect | + | + | *Bacillus* sp. |
| 36 | GK_GR_104 | YMA | White | Glossy | Translucent | Spread | Viscous | Normal | + | ++ | No Match |

**Figure S1**

**
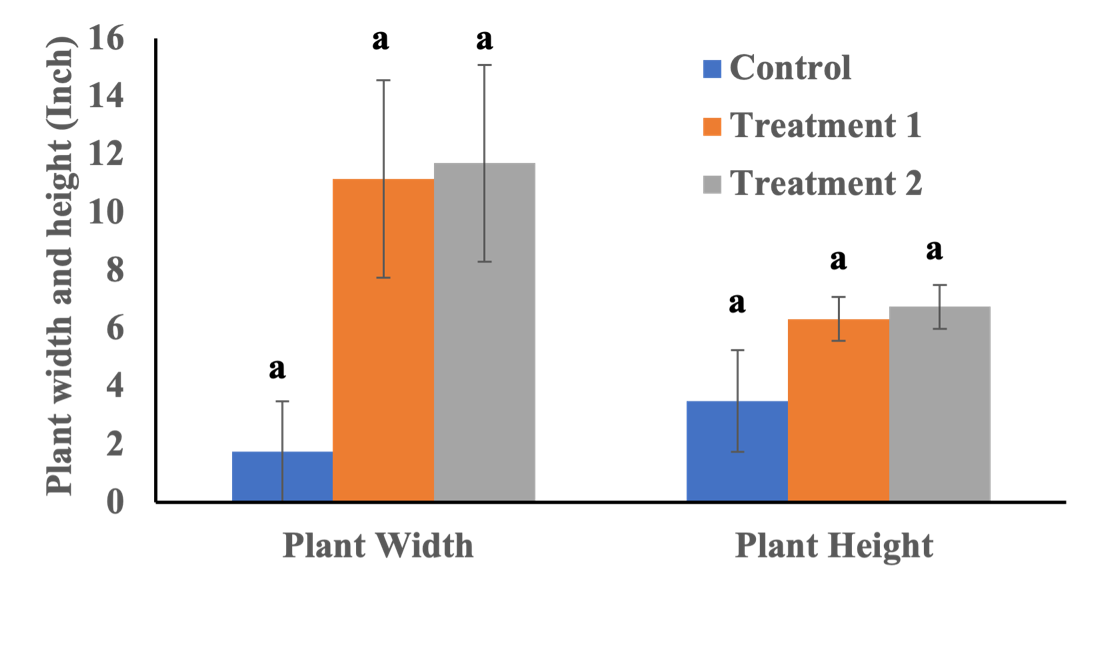
**

**Figure S1**Measurement of plant width and height of snowbrush ceanothus plants (with and without native soil) after six months. The plants treated in treatment 1 (native oil from elevation 1950m AMSL) and treatment 2 (native soil from elevation 2289m AMSL) showed bigger plants compared to control plants not treated with native soil. The pot diameter is 16cm. No significant difference has been observed between the control and treatment. The same letters denote no significant difference among treatments by Tukey's method for multiplicity at α < 0.05.

**Figure S2**

**
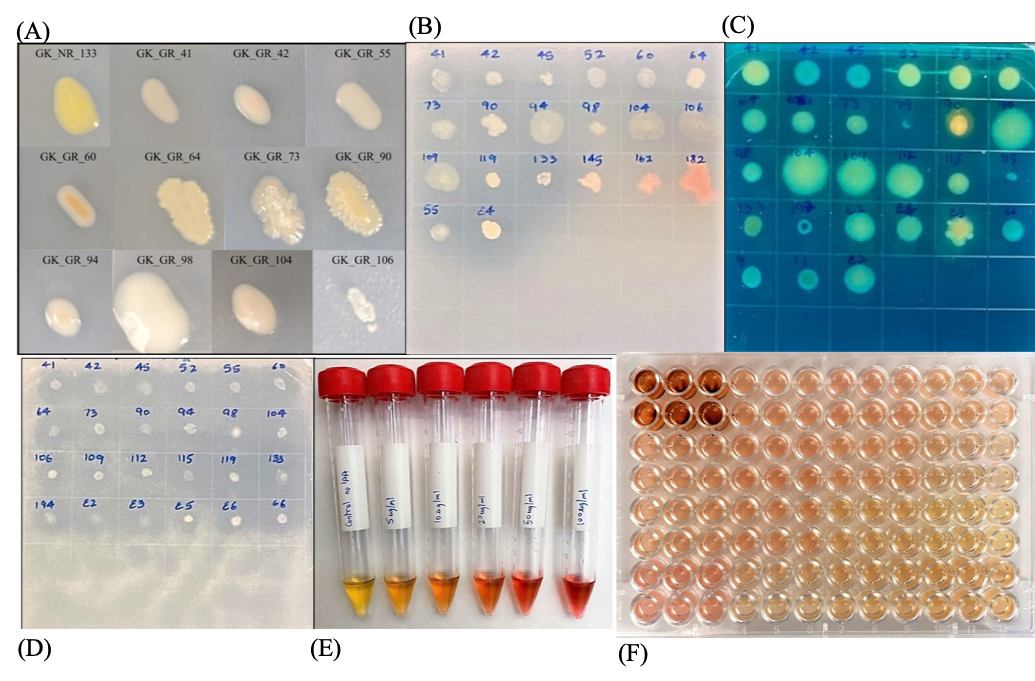
**

**Figure S2 (A).** Bacterial characterization of twelve colonies that tested positive for all the PGP traits. (**B)** Phosphate solubilization in Pikovakaya’s agar media. **(C)** siderophore production in CAS agar media. (**D)** Nitrogen fixation in Norris Glucose Nitrogen-free media. (**E)** IAA standard, and (**F)** IAA production.

**Figure S 3**

**Figure S3** The Standard curve for IAA was created for 5,10,20,50, and 100 µg/ml of purified IAA (Sigma Aldrich) and read at ABS_530nm_, and an equation was used to calculate the IAA concentration for unknown samples.
